# Primary School Educators’ Perspectives and Experiences of Nature-Based Play and Learning and Its Benefits, Barriers, and Enablers: A Qualitative Descriptive Study

**DOI:** 10.3390/ijerph19063179

**Published:** 2022-03-08

**Authors:** Nicole C. Miller, Saravana Kumar, Karma L. Pearce, Katherine L. Baldock

**Affiliations:** 1 Clinical and Health Sciences, University of South Australia, 108 North Terrace, Adelaide, SA 5001, Australia; karma.pearce@unisa.edu.au; 2 Allied Health and Human Performance, University of South Australia, 108 North Terrace, Adelaide, SA 5001, Australia; saravana.kumar@unisa.edu.au (S.K.); katherine.baldock@unisa.edu.au (K.L.B.)

**Keywords:** nature play, nature-based learning, primary school, enablers, barriers

## Abstract

Nature-based play and learning is of increasing interest to primary schools and research suggests that it has many potential benefits for children’s health and development. However, little is known about educators’ perspectives and experiences of nature-based play and learning, particularly the barriers, benefits and enablers, despite their direct relevance to the uptake of nature-based play and learning in schools. A qualitative descriptive methodology was employed to uncover these. Individual, semi-structured interviews were conducted with 12 principals and educators from South Australian public primary schools, recruited via a participant contact list from a previous study. The participants were two principals, eight educators and two individuals with dual principal and educator positions. Metropolitan and rural schools were equally represented. Interviews were audio-taped, transcribed verbatim and analysed using thematic analysis. Analysis identified four overarching themes: the practice, perceived benefits, barriers and enablers of nature-based play and learning. Children’s learning, enjoyment, creativity, and a relaxed and flexible environment were clear benefits. Meanwhile educator knowledge and confidence and the crowded curriculum were barriers. Enablers were nature-based play and learning champions and support from school leadership. The findings suggest that schools can help engage students with nature-based play and learning activities by mitigating these barriers and promoting these enablers.

## 1. Introduction

The interest in both nature-based play and nature-based learning appears to be growing in primary schools across the globe [1,2,3,4]. While there are no universally accepted definitions, nature-based play is unstructured play with nature [5], and nature-based learning is the use of nature and natural items to enhance learning across the curriculum, inside or outside of the classroom [4,6]. The interest in nature-based play and learning in recent years is likely to be due to the increasing evidence and awareness of the potential benefits that nature-based play and learning can have for children. These include benefits to learning, engagement, physical activity, social skills and mental health and wellbeing [7,8,9,10]. This suggests that nature-based play and learning has potential as a health promotion tool [8,11], particularly within the school environment, which can be a powerful setting for influencing behaviour [12,13]. With this increasing evidence and interest in nature-based play and learning in schools, it is essential to understand the perspectives and experiences of school staff. Children’s outcomes and experiences of nature-based play and learning have been the focus of previous research [7,8,9,10] and few studies have investigated staff perspectives [4]. School staff are the providers of children’s experiences at school and thus directly impact the uptake of nature-based play and learning in their schools. Understanding their perspectives and experiences of nature-based play and learning, including the benefits, barriers, and enablers can help to inform future interventions to increase uptake and improve the practice of nature-based play and learning.

Previous research into school staffs’ perspectives and experiences of nature-based play and learning has included surveys [2,14,15,16,17,18,19], focus groups [20,21], and interviews [4,17,18,21,22,23,24,25]. The findings of these studies suggest that the most prevalent benefits perceived by school staff include improved social skills [2,4,14,22], health and wellbeing [4,14,23], connection to and awareness of the environment, physical health and development [2,14], learning [2,22] and engagement with learning [4,22]. Other perceived benefits identified in the literature include improved cognitive development [14], critical thinking [24], improved behaviour [4], community connectedness, life skills [2], teacher-pupil relationship [24], broader educational experiences [25], teacher motivation [22], and job satisfaction [4].

However, this research also reports that barriers interfere with the implementation of nature-based play and learning within schools. The most prevalent barriers were limited access to nature or a suitable outdoor space [4,14,15,16,17,19,20,21], limited time [4,15,16,18,19,20,21], lack of teacher knowledge and confidence [4,17,18,21,22], lack of resources (including funding and materials) [4,15,16,21,23,25], curriculum demands (including standardised testing and evidencing work) [4,21,23], safety concerns [4] and lack of support from colleagues [25]. Understanding these barriers is important in implementing and increasing nature-based play and learning uptake. 

Understanding the enablers of nature-based play and learning is valuable to combat these barriers. These enablers are any factors that make it easier for school staff to use nature-based play and learning. However, just two studies have investigated enablers [4,21]. These studies were both conducted in the United Kingdom (UK). One study used interviews and focus groups with 68 school staff from 12 schools [21] and the other interviewed 13 upper-primary teachers using outdoor learning [4]. The enablers reported by previous research include school leadership that is supportive of the use of nature-based play and learning, and volunteers that assist in the supervision of nature-based play and learning [4,21]. One additional study that interviewed schools that had developed green schoolyards found that they enabled nature-based play and learning and mitigated barriers such as costs for travel, time constraints and additional support [17]. The limited information on the enablers of nature-based play and learning is a key research gap and further research is required to understand the enablers of nature-based play and learning in primary schools.

When seeking rich perspectives and experiences, qualitative research is most appropriate [26]. The current body of research presented above includes a handful of qualitative studies [4,17,18,20,21,22,23,24,25]. Along with the two studies reporting enablers described above, these studies included one study that used focus groups with 13 staff at an after school programme incorporating environmental education in the United States and several studies that interviewed various groups including 21 primary school staff from 5 Canadian schools about outdoor learning [17], 26 primary school teachers in Queensland about environmental education [18], 12 high school teachers from Sweden using outdoor learning [22], 8 primary school teachers in Denmark using udeskole (outdoor school) [23] 9 high school teachers from 3 schools in Scotland using outdoor learning [24] and 10 primary school teachers in Denmark using udeskole [25]. These previous qualitative studies have investigated the perspectives and experiences of school staff on environmental education, LINE, outdoor learning and udeskole and thus provide some insight into the perspectives and experiences that may be relevant to nature-based play and learning. However, several knowledge gaps remain. First, to date, no studies have investigated school staffs’ perspectives and experiences of nature-based play and learning. Second, these studies have mostly been conducted in European countries, including the UK [4,21,24], Sweden [22] and Denmark [23,25]. No studies have investigated the perspectives and experiences of nature-based play and learning in the Australian context. School staffs’ perspectives and experiences are likely to vary depending on the cultural and geographic context; thus, it is important to investigate these in a variety of contexts. There is also a dearth of research on the enablers of nature-based play and learning; such evidence is critical to inform interventions to increase its implementation within the primary school setting. Finally, no research has captured school staffs’ perspectives and experiences of nature-based play and learning during the COVID-19 pandemic. Therefore, this study aimed to address these gaps by gaining a richer understanding of school staffs’ perspectives and experiences of the benefits, barriers and enablers of nature-based play and learning in an Australian context. This study will focus on primary (elementary) schools that cater for children aged 4 to 12 years.

## 2. Methods

### 2.1. Study Design

To align with the aim of gaining a rich understanding of school staffs’ perspectives and experiences of the benefits, barriers and enablers of nature-based play and learning in an Australian context, a qualitative descriptive methodology was chosen. Qualitative descriptive research is ideal for studies that aim to provide straight descriptions of phenomena, with the aim being to describe phenomena of interest comprehensively and keeping closely to the words and meanings of participants. This is done by focusing on keeping the information as close as possible to what was intended by the participant and presenting information in everyday language similar to the participants’ own words [27,28]. This research was conducted in accordance with the best practice for reporting qualitative research, the COREQ Checklist (see Supplementary Material S1) [29].

### 2.2. Study Sample

The sampling frame for the current study was a participant contact list from a survey conducted by the authors in 2019 that investigated the practice of nature-based play and learning in primary schools. The contact list consisted of 28 survey participants who had expressed their interest in participating in a follow-up interview. The survey recruitment strategy sent recruitment emails to the generic email addresses of all South Australian public primary schools (n = 427). The survey was also promoted to educators in the newsletter of Nature Play SA, a local organisation that promotes nature-based play and learning to schools. To be eligible to participate in the interview, participants needed to be educators or principals at South Australian public primary schools. In the current study, non-probability, self-selection sampling was used [30]. Participants were contacted via the email address they voluntarily provided. The recruitment email included a brief introduction to the study and the interviewer (name, role and research interests), and a detailed participant information sheet was attached. Additional follow-up emails were sent at two, four and six weeks after initial contact. In qualitative research, there are no recognised sample size standards [31]. Therefore, the sample size was justified using the guidance of qualitative methodologists [31], previous research [4] and ensuring that the data explored all areas of interest [32]. While additional interviews were planned, they were not required as all relevant areas were covered. 

### 2.3. Data Collection

Data were collected by one-off, one-on-one, semi-structured interviews with participants. This method was chosen to allow for free-flowing and open conversations with space for elaboration and clarification [32,33]. The interviews were conducted using an interview guide (see Supplementary Material S2), which was produced and piloted within the research team and included a set of questions and prompts. This guide was a tool for the researcher and was not provided to participants. The interview questions centred around the use of nature-based play and learning at school and participant experiences of the barriers, benefits, challenges, and successes, as well as perspectives of the available resources. Demographic information had already been collected as a part of the previous study (the NaPSA survey). This information included age, gender, role, years of experience, location and Index of Educational Disadvantage (IED). IED is a measure used by the Department for Education to classify the disadvantage of the populations that schools serve (1 = most disadvantaged and 7 = least disadvantaged) [34]. The interviews were conducted via telephone and lasted between 25 and 55 min. Telephone was chosen over teleconferencing as this method was considered less of a burden on participants, was most accessible for participants who may not have access to teleconferencing equipment and as only audio data was required. The interviews were audio-taped, and no field notes were taken. Participants were informed that the interview would be audio-taped on the participant information sheet and gave their consent for this on the consent form prior to the interview. During the interview participants were informed when they were being recorded. In qualitative research, a researcher’s values and interests can play a role in influencing data collection and analysis [32]. It is important to reflect on these. The interviewer was one member of the research team (N.M.), a female PhD student with growing expertise in nature-based play and learning practice and research. The interviewer was aware of her personal beliefs as a supporter of nature-based play and learning and regularly discussed these with the research team. The interviewer had some qualitative research experience and no prior experience conducting interviews. Thus, she was guided by the research team, who have extensive knowledge and experience in qualitative research and interviews. At the beginning of the interview, participants were reminded that the research aimed to collect their true perspectives and experiences and that there were no right or wrong answers. The researchers had no prior relationship with the participants.

### 2.4. Data Analysis

The interview audio-tapes were transcribed verbatim into NVivo 12 plus [35]. Thematic analysis was employed by one coder (N.M.) to analyse the data. Another research team member (S.K.) checked the analysis, and any disagreements were resolved via discussion. The thematic analysis involved deriving themes from the data by detecting patterns across the data set and classifying these patterns into themes and sub-themes following a six-phase procedure [32]. First, the data were read and re-read to establish familiarity. Next, codes (labels) were derived from possible patterns identified in the data. Following this, similar codes were grouped into larger possible patterns (potential themes and sub-themes), then compared with the dataset to ensure they were representative. Next, a descriptive name and definition was developed for each theme and sub-theme [32]. Participants were given the option to review a copy of their transcript. After analysis, participants were sent a summary of the results and given the opportunity to provide feedback. However, no participants requested a transcript or provided any feedback. Throughout the data collection and analysis, rigour was upheld using several methods. Overall, the thematic analysis procedure was carefully followed, and the analysis was checked to ensure that the codes, themes, and sub-themes were a genuine reflection of the participants’ responses in the interviews. Credibility was maintained with the use of a second coder and persistent observation of the data. Transferability was upheld with the use of thick, rich description of the participants and methods [32]. Dependability was maintained through the keeping of records describing the research steps taken. Finally, confirmability of the data was maintained through the researchers’ continuous awareness of their individual biases and prior assumptions and the study’s limitations [36].

### 2.5. Ethics

This research was approved by the University of South Australia Human Ethics Committee (Application ID: 202848) and the Department of Education (Reference No: 2020-0015). Participants were provided with a participant information sheet that included information about the purpose of the research and what was required. The sheet also stated that their participation was voluntary, and they could withdraw at any time without consequence. Participants agreed to participate by signing a consent form prior to the interview.

## 3. Results

Of the 28 participants who expressed interest, 12 participants consented; 8 were educators, 2 were principals, and 2 held dual roles as principals and educators. Of those who did not participate, 11 did not respond, and 5 declined. Participants declined as they were not or were no longer in an educator or principal role (n = 2), did not have the time (n = 2) or did not feel that they had enough knowledge on the subject (n = 1). 

Most participants were aged 45 to 54 years (42%), female (92%), with 20 or more years of experience in the school environment. Participants’ schools had varied Index of Educational Disadvantage rankings, with most being advantaged (index 5–7), and equal proportions of participants were from rural and metropolitan schools. The demographic characteristics of participants are displayed in Table 1. Thematic analysis of the interview data identified four key themes; (1) the practice of nature-based play and learning, (2) perceived benefits, (3) perceived barriers, (4) enablers and a number of sub-themes.

*Theme 1: The practice of nature-based play and learning in primary schools*

The first major theme identified included information about the daily practice of nature-based play and learning in participants’ schools and the spaces in which it took place. There were three key sub-themes identified.

Sub-theme 1: The growing interest in nature-based play and learning in primary schools

Several participants described the increasing popularity of nature-based play and learning in primary schools and the growing interest in engaging students with nature. This popularity appears to be an important element in the practice of nature-based play and learning in primary schools. This is exemplified in the following comment made by a participant:

“… there is just more exposure to nature play now… there is more articles educating people on the benefits of it why we do it and… we are continually talking about it because it is coming up in all of our class meetings…”

(Participant 6, educator)

Some more experienced participants explained how the popularity and perspectives of nature-based play and learning had shifted throughout their careers. This shift was described as occurring in the school rules, and in the transformation of school yards to include more natural elements. Participants described that some activities, such as play with sticks and tree climbing, had been considered inappropriate but were becoming more common. One participant described the change in perspectives concerning the rules for stick play over their teaching career:

“…so when I first started teaching, …you could play with them, and then sticks were banned, you could not pick up a stick, lift a stick, walk with a stick, do anything with a stick. So now it’s kind of like they’re just always- like there will be 2 or 3 of them on a branch shifting it to the next spot that they want it”

(Participant 10, educator)

Most participants’ schools allowed children to climb trees, and often this was a relatively recent change in the school rules. Most participants reported that there were rules for the height to which students were allowed to climb. One participant described their experience:

“Yes! Since I’ve been here… we do let the kids climb the trees to a certain level in our jungle area… and they all value that, all the teachers value that, but we do still have to convince the parents that that’s OK because they could be afraid of risk-taking, and they’ve taken a bit [of encouragement] to get on board…”

(Participant 6, educator)

One participant described the evolution of the tree climbing rules at their school and how the height rules were no longer required:

“…we used to have a red spot on all the trees that they were allowed to climb, and as long as their feet were above that spot, they were allowed to climb those trees, but now we’ve eliminated that, it’s just like you climb a tree until you feel comfortable or the staff member supervising you feels comfortable.”

(Participant 10, educator)

The increase in popularity of nature-based play and learning was also reflected in the elements included in participants’ schoolyards. Participants discussed the evolution of play spaces and schoolyards to include more natural features and green space. One participant shared their experience:

“Yeah, it is changing, there is more focus of going and playing outside and going from more metal structures… to more natural wood, wood structures and play structures to make it more natural and ensuring that children are having opportunities to work as a group…”

(Participant 11, educator)

Sub-theme 2: The spaces where nature-based play and learning are practiced

There was a focus on the spaces where nature-based play and learning took place as an important aspect of its practice. All participants’ schools had purpose-built nature-play spaces in their schoolyards. Purpose-built nature play spaces are human-made spaces in schoolyards created to engage children with nature-based play and learning. Participants provided descriptions of the features present in their purpose-built nature play spaces. Common features in these spaces included trees, dry creek beds, kitchen gardens, and logs and rocks, which are often used for climbing and as seating areas for outdoor classes. Although these features were common, a wide range of spaces were described. Some participants described spaces that were designed and constructed by professionals, such as in the following description from one participant:

“We’ve got ropes, like net area where they can climb, a climbing area and with nets and sand…, they’ve got mounds, they’ve got bridges, they’ve got those big rocks, you know sitting rocks, shade structures, they’ve got poles, those big, long poles, they’ve got a water feature...”

(Participant 11, teacher)

While some participants described much simpler spaces, in some cases, these were designed and constructed by members of the school community. One participant described how the school community had designed and created their space themselves:

“We already have a large sort of free form sandpit, one of the dads brought his digger in one day… [our space is] not planned by anyone professional and very simple—rocks, bushes and a couple of... concrete drainpipes dug in…”

(Participant 5, educator)

Sub-theme 3: Focus on practicing nature-based play and learning with junior primary students

Several participants described a variety of experiences of practicing nature-based play and learning with their junior primary students. In contrast, for upper primary students, the use of nature-based play and learning was limited. Some participants explained the perception that nature-based play and learning is more suitable for junior primary students and that upper primary students should be spending their time in traditional classroom-based learning, such as the following:

“I think that’s where early years leads to nature play; it’s because it’s got that ‘play’ word in it there, and that’s what everyone gets afraid of, like, “Oh, you’re not in junior primary anymore, so you can’t play”.”

(Participant 6, educator)

The following statement by a principal corroborated this:

“…because of curriculum expectations, people see that as more sat down at a table once they get to the older years rather than going outside and using the nature to incorporate that into their lessons, so I think it’s the expectation of teachers and probably parents as well.”

(Participant 12, dual principal and educator)

*Theme 2: Perceived benefits of nature based play and learning in primary school*

Participants described a number of benefits that they perceive and experience when engaging their students in nature-based play and learning. The following four key sub-themes were identified.

Sub-theme 1: Relaxed and flexible environment

A theme running through the participants’ perspectives of the benefits of nature-based play and learning was that nature-based play and learning could provide a more relaxed and flexible learning environment. Participants described how this environment was beneficial as it helped some children to feel calmer. Participants reported that they believe this is due to the reduction of the pressure and structure that is usually present in a traditional classroom environment:

“…they’re more relaxed because it’s not that structured learning—”everyone sit on the floor”, explicit instruction, pressure pressure pressure—it’s problem-solving, it’s finding out consequences, actions, it’s thinking, coming up with ideas themselves. It’s not: classroom, me talking, them racking their brains to work out what has to be said saying the right thing, not understanding what’s going on…”

(Participant 9, principal)

Furthermore, some participants discussed how the more relaxed setting that nature-based play and learning can create can also help to boost the confidence of some children:

“…there are a lot of kids that are frightened of putting their hand up to answer a question in case they get it wrong, but it’s different outside… and there are some specific kids that I am thinking of that are afraid to open his mouth in case he got the answer wrong but outside—different altogether, completely different.”

(Participant 4, educator)

This experience was also shared by principals, as described by this participant:

“…there is other children who just do not cope in classrooms anymore. When they get out into the [outdoor] space they can demonstrate their creativity, their knowledge, their communication because they are not under the academic pressure of classroom behaviour—they can be outside in space, so we see very little negative behaviours in our space in nature play...”

(Participant 9, principal)

Participants described that this environment also had a positive impact on educators by allowing them to be more flexible in their teaching:

“But also, it’s kind of cool because it gives you also unpredictability, and that’s really nice as a teacher, to do things that aren’t exactly mapped out.”

(Participant 8, educator)

Sub-theme 2: Real-world learning

Several participants discussed the benefits of nature-based play and learning for children’s learning. Participants felt that nature-based play and learning provided more opportunities for ‘hands-on’ and real-world learning and that this helped children to be engaged in class and feel like what they were learning about was relevant to their lives. This is exemplified by the following comments by participants:

“…if they are doing things outside, they can see the purpose why we are learning things and we can see that learning is not just in the classroom—like, they are learning every day.”

(Participant 6, educator)

“…it just feels a little bit more meaningful… like problems and things like that that actually have something to do with life. Like a lot of our maths learning going around the garden and the kitchen where the food is collected and whatever else you know it’s the sort of learning that I think kids will find meaningful, engaging and it’s something that I think will probably stick with them”

(Participant 8, educator)

Principals also shared this perspective:

“It’s more engaging for the kids to go out and actually be hands-on and learn about something relevant to them in their own backyard... So, it definitely promotes student engagement and learning.”

(Participant 1, deputy principal)

Sub-theme 3: Opportunities for creativity

Another key benefit discussed by participants was the opportunities that nature-based play and learning could provide for children to be creative in their play and learning. Participants discussed the open-ended nature of the outdoors and the opportunities for creativity that this can provide.

“…they can do stuff in different ways, there’s more things to explore rather than just inside when they’ve just got plastic toys or whatever… [when] they’re outside… they can use stones, they can use pebbles, they can use sticks… it’s giving you a wilder experience.”

(Participant 12, assistant principal and educator)

“Just the freedom… they don’t feel restricted and there is not, like, the one way to do things…”

(Participant 6, educator)

One participant described how the opportunities for creativity are also enhanced for educators when using nature-based play and learning:

“I reckon it’s the creativity and imagination because… my colleague and I, we go out with this idea in mind and then suddenly we are like, “Oh hang on, they’re [the children] doing that, but hang on, that’s really cool…””

(Participant 10, educator)

Sub-theme 4: Enjoyment

The final key sub-theme of the benefits of nature-based play and learning was enjoyment. Many participants highlighted the excitement and enjoyment that many students get from engaging in nature-based play and learning:

“…it doesn’t matter what year you teach—R-7s [Receptions to year 7s] they just enjoy being outdoors”

(Participant 3, educator)

“The kids love it; the kids love any opportunity to get outdoors really…”

(Participant 1, principal)

Furthermore, some participants explained that it is not only enjoyable for the students but also for the staff:

“I think they [the staff] enjoy it, so last term we were doing the project on shapes; all the teachers were laughing with the kids and enjoying being out there… it was just fantastic- it’s exciting, and it’s motivating.”

(Participant 2, principal and teacher)

*Theme 3: Perceived barriers to nature-based play and learning in primary school*

It is evident that participants perceive a number of valuable benefits of nature-based play and learning at school. However, there are also a number of barriers that hinder the use of nature-based play and learning. These barriers have been categorised into the following five sub-themes.

Sub-theme 1: Educator openness, knowledge and confidence

A key barrier experienced by participants was educator openness, knowledge and confidence. Participants described how some educators felt they lacked the knowledge or the confidence to take their students outdoors, and thus they were less open to trying nature-based play and learning with their students. This is shown in the following quotes from participants:

“…just getting out of their comfort zone in the classroom is a barrier for a lot of teachers.”

(Participant 1, deputy principal)

“…it’s having that confidence to think… I’m still covering curriculum, still doing what I need to do, but I’m just doing it in a different creative way, having the confidence to say that and actually do it.”

(Participant 12, dual principal and educator)

In some cases, this lack of confidence seemed to be closely connected to fears of losing control of the class:

“I think some teachers might feel safer with four walls around them and feel like they have got that comfort of everything that they need, or this is about maybe their perception that everything that they need, like, whether it’s maths counters, or flashcards, or whatever, it’s inside their four walls and… if they go outside, it might go a bit crazy.”

(Participant 3, educator)

“There are issues with teaching in the outside and I know this but they’re not… insurmountable. It’s very convenient to sit inside with your electric whiteboard and your controlled things… I think the biggest issue with teachers is control…”

(Participant 7, educator)

Sub-theme 2: Crowded curriculum

Participants described the crowded curriculum as a barrier to nature-based play and learning. The crowded curriculum refers to the expectations on educators to educate students about a growing number of social issues such as sustainability, consent and cultural awareness. When combined with literacy and numeracy and the pressures of standardised testing, the curriculum becomes very full. Participants described the immense pressure on educators to meet the many expectations and demands upon them:

“…the curriculum is so crowded and the pressure on teachers is absolutely phenomenal… teachers and principals are under a lot of pressure to create data.”

(Participant 7, educator)

“…we are accountable for a lot of things and we do need to make sure that we are covering all the curriculum areas… life just gets really busy at school and sometimes it happens and sometimes it doesn’t and you have these little unexpected occurrences that pop up during the day…”

(Participant 3, educator)

Principals also corroborated this:

“Yes, the curriculum is so jam-packed and so data orientated that you know if you don’t just make time then it doesn’t happen… the curriculum and the demands are ridiculous.”

(Participant 9, principal)

Sub-theme 3: Funding

Several participants described funding as a barrier to nature-based play and learning. However, this barrier had varying degrees of impact upon participants. One participant described how expensive items for purpose-built nature play spaces could be:

“…the things that we did purchase… they were really expensive, like the thing that they balance on… they’re just bits of wood, but they did cost a lot, the kids probably enjoy climbing over a big log or tree stump just as much.”

(Participant 3, educator)

Several participants had applied for grants to fund parts of their schoolyard development. One participant shared how they were unsure how to access grant funding:

“…but we are trying to get some funding, it’s just trying to find the right one and often what we want it is not a lot of money, and just trying to word it and also trying to find where to go for that…”

(Participant 6, educator)

On the other hand, some participants had successfully navigated with little to no funding:

“…we have taken a piece of area in the school, and we have created a nature play area, I would love to have 10 to 15 thousand dollars to create a… purpose-built area… but we haven’t… so we have got some slopes, we have got some flat area, we have got an area where we have created a pretend river bend… So, we have got a shoestring budget obviously, but we try and create what we can...”

(Participant 9, principal)

“No, no money whatsoever, the space was there… it was a sheep paddock, from our Ag[ricultural] area and it was all moved, weeded, things moved around—my husband had got heavy machinery—on weekends through working bees and things like that.”

(Participant 4, educator)

Sub-theme 4: Weather

A key barrier to nature-based play and learning at school was the weather. Participants discussed how bad weather can derail their plans despite their intentions to engage in nature-based play and learning. One participant described the difficulty in finding suitable times to play and learn outside among hot summers and cold winters:

“…in summer we do have a snake problem here, so when it’s hot we’re not outside… And then in winter, it is very cold, so you’ve got to find those balance days, and we try to use those as much as we can.”

(Participant 2, principal and educator)

However, participants highlighted that this often was not a concern for the students.

“It is like at times it feels harder going, because, like, if it’s a bit windy or sometimes it gets a bit heated or a bit chilly so we’re standing there and we’re like, “Oh you want to do this?” and you turn to the kids, and they’re like, “Yeah! Let’s go!””

(Participant 10, educator)

“I said to them, “Who’s wet?”, and they all put their hands up, and “Who cares?” and they all shoot their hands down.”

(Participant 7, educator)

Sub-theme 5: COVID-19

Many participants discussed the impacts of COVID-19 on their opportunities to engage students in nature-based play and learning. Participants reported that COVID-19 had interrupted a number of nature-based play and learning events, excursions, incursions, activities, training and activities that required parent volunteers. In addition, some participants described that the pressures of the crowded curriculum were compounded by the impacts of COVID-19 restrictions, making it more difficult to find time for nature-based play and learning:

“… sometimes the teachers will say to me they’ve got too much on their curriculum, trying to get through all of their curriculum stuff, especially now with this COVID stuff that there is a lot of home learning, teachers are feeling that they’ve missed out on lots of explicit learning…”

(Participant 11, educator)

On the other hand, some participants found that the impacts of COVID-19 did not affect nature-based play and learning at their school:

“COVID-19 stuffed up many other things but not nature play.”

(Participant 9, principal)

Some participants described how their traditional playgrounds were closed or regularly cleaned, while the purpose-built nature play and natural outdoor spaces remained open:

“…the only area that we did close off was the playground equipment, and that then was being wiped down daily and is open to the children… our nature play and forest went on as usual, wasn’t impacted at all...”

(Participant 11, educator)

*Theme 4: Enablers*

Participants also shared the enablers that make it easier to use nature-based play and learning and help in mitigating the effects of some of the barriers.

Sub-theme 1: A nature-based play and learning champion

A key enabler was the presence of a champion; a champion is someone, usually an educator, who advocates for nature-based play and learning at their school. One participant who had a nature-based play and learning champion at their school said:

“Find a key staff member, so if someone is really passionate about it who is prepared to take on… a lead role and find out information and access grants and things like that—so, like, what we did.”

(Participant 1, deputy principal)

In one case, a participant described the impact of their nature-based play and learning champion on their school. This champion had cultivated the use of nature-based play and learning within the school and schoolyard. However, when this champion left the school, some behavioural issues arose within the purpose-built nature-play space without her influence:

“We had a teacher here… absolutely passionate about using the outside area and… had that embedded into her teaching whether it be maths, literacy, whatever, and she left, and so since then… children are not using it in the way we intended it to be used… So, lots of fighting with sticks and stuff like that.”

(Participant 4, educator)

Sub-theme 2: Support from leadership

The second key enabler of nature-based play and learning discussed by participants was support from leadership. Participants describe that using nature-based play and learning was much easier if school leaders such as principals and governing councils supported it:

“…we are quite encouraged to take learning outside… we often have staff meetings and we discuss how are we doing maths outside and how are we doing literacy outside and that’s just there to inspire us a little bit more

(Participant 8, educator)

“…you need people as passionate… we’ve got two teachers that are passionate about it—they seem to run with it, but it needs to be… programmed by a whole school.”

(Participant 11, educator)

One participant described their experience with their principal:

“…yeah, she’s [the principal] really supportive… if your leader wasn’t- I guess you do it as a class teacher, like, I just decided as a class teacher to do outdoor classroom day, but it’s nice when your whole school is kind of that way inclined.”

(Participant 10, educator)

Sub-theme 3: ‘…just go outside, just get out there!’

Finally, participants indicated that nature-based play and learning could be successfully undertaken using existing resources, access to everyday tools, and simply taking opportunities to get out into nature. One prevalent idea was that the setting for nature-based play and learning does not need to be perfect. Participants described how although they would like one, they did not need the perfect space to engage students in nature-based play and learning successfully:

“…I think it’s about what value you put on it and if you had a tiny space with some pot plants in a concreted area you would find ways to get outside and make it work.“

(Participant 9, principal)

“…you don’t have to spend a fortune—keep it small, you could even get small car tyres free and just use them as planters, or use them as digging pots for children with some spades or wooden spoons, or pots and pans from the jumble sale—you know, it doesn’t have to cost a lot of money to create an outdoor space.”

(Participant 12, dual principal and educator)

“I think: number one, nature doesn’t have to be going to a forest, it could be just something that’s outside your classroom, and you can use it every day, and it is there to help you teach—use it as a tool rather than a hindrance...”

(Participant 8, educator)

Another message shared by participants was to take opportunities to engage in nature-based play and learning and not be afraid to try it out with students.

“…just go outside, just get out there! Just try it.”

(Participant 3, educator)

“I’d recommend that teachers take up the opportunities to teach outside the classroom as much as they can.”

(Participant 11, educator)

## 4. Discussion 

This study aimed to uncover the perspectives and experiences of South Australian public primary school educators about nature-based play and learning. The findings provide insight into the everyday practice of nature-based play and learning and the perceived benefits, enablers, and barriers encountered by educators. This research has made a novel contribution to the literature in the following ways: (1) this was the first study to investigate educators’ perspectives of nature-based play and learning in an Australian context; (2) this study contributed to the limited body of evidence that has investigated nature-based play and learning from the staff perspective rather than the student perspective; (3) this study provided insight into the enablers of nature-based play and learning that have previously been investigated by only two studies; and (4) this study collected educators’ perspectives and experiences of nature-based play and learning during the COVID-19 pandemic. The findings of this research indicate that for nature-based play and learning to become embedded as part of the school day, barriers need to be mitigated through enabling strategies so children can access the potential health and wellbeing benefits. 

The current study highlights the growing interest in nature-based play and learning in primary schools. This growing interest has also been reported by previous studies [1,2,3,4]. As shown in the sub-theme ‘the growing interest in nature-based play and learning in schools’ this interest is an important element in the practice of nature-based play and learning in schools. As described by participants this growing interest appears to have resulted in changes to schoolyards and educator perspectives of nature-based play and learning. Schoolyards appear to be transitioning to incorporate more greenspace and include purpose-built nature play spaces. This finding was also reported by previous research [4,37]. 

The findings of the current study highlight the focus and importance that South Australian educators place on purpose-built nature play spaces for the practice of nature-based play and learning. The descriptions of purpose-built spaces in participants’ schoolyards varied widely from spaces designed and built by the school community to professionally designed and constructed spaces. This may suggest that there is no one-size-fits-all solution and schools may choose bespoke approaches to developing nature-based play and learning spaces to suit their local needs and requirements. However, it is unclear what effect different spaces have on children’s outcomes. Perspectives of risky play also appear to be changing. Risky play activities such as tree climbing which may have previously been viewed as unsafe, were now reported by participants as an important aspect of nature-based play and learning. Perhaps this is a result of research that shows that risky play can aid in children’s development [38,39,40]. Research also suggests that risky play provides children with opportunities to discover and expand their cognitive and physical boundaries [40,41]. It is important to note that risky play does not mean that safety is disregarded; instead, hazards are eliminated without removing the opportunities for children to experience risk and challenge [42]. 

The benefits of nature-based play and learning described by participants in the current study were primarily centred around the unique learning environment that nature affords. Participants described how this learning environment can be less structured and put less pressure on students than a traditional classroom environment. Some participants described how this environment can help boost some children’s confidence in a group learning context. This finding was reflected in a case study of high school teachers using nature-based learning. Teachers reported that more students, particularly shy children, actively participated in class during outdoor learning [22]. The perception that nature-based play and learning can benefit learning has been identified in previous studies [2,4,14,22]. In addition, previous research has identified opportunities for creativity [4] and enjoyment as perceived benefits [14,22]. These findings suggest that the unique environment created by nature-based play and learning may be beneficial for students and thus is an important asset to schools.

Despite the movement towards nature-based play and learning, this research also identified a perception among educators that it is less suited to upper-primary school students. This finding is reflected in a study from Denmark that found udeskole (outdoor learning) was used primarily in the early years [43]. Similarly, a survey of 334 settings (such as pre-schools and primary schools) reported a decline in outdoor learning activities after the early years [44]. The authors of both studies suggest that this may be because there were fewer competing academic priorities in the early years [43,44], which may change in later school years. This corroborates with the finding that the crowded curriculum is a significant barrier to nature-based play and learning. 

In terms of the barriers, the findings reported in this research are mirrored by previous studies. The sub-theme of educator openness, knowledge and confidence was reported in several other studies [4,17,18,21,22]. Also commonly reported were crowded curriculum [4,16,18,21,23], lack of funding [4,15,16,21] and weather [4,17,19,21]. It is clear from the evidence base that these barriers are experienced across a wide range of schools from various geographic and socioeconomic contexts. This suggests that interventions that mitigate barriers and support enablers could positively impact the uptake and use of nature-based play and learning in schools. Staff training to increase knowledge and confidence in using and integrating nature-based play and learning into the curriculum may be one effective strategy. 

Given the timing of this research in relation to the COVID-19 pandemic, COVID-19 has not been identified as a barrier to nature-based play and learning in previous research; this is likely due to its recency. Participants reported varied impacts of COVID-19 on nature-based play and learning, including increased use. This suggests that the impact of COVID-19 on nature-based play and learning varied widely across settings. Research indicates that COVID-19 has disrupted education in an unprecedented way [45]. According to the United Nations, closures and interruptions from COVID-19 have impacted 94% of students globally [46]. 

In order to allow students to access the perceived benefits of nature-based play and learning, the barriers need to be mitigated, and the enablers need to be promoted. The current study identified a nature-based play and learning champion as a key enabler; this has not been previously identified. However, the use of change champions is a strategy often used for implementing change in a variety of organisations. Change champions are individuals who start and follow through with change in their organisation [47]. A recent review of change champions in healthcare identified that change champions were consistently associated with implementation success [48]. 

The second key enabler reported was supportive leadership. One previous study of 119 staff at South-West England primary schools also reported support from leadership as an enabler [21]. As the participants of this study described, educators face immense pressure to create data and meet curriculum demands. The presence of school leaders who back nature-based play and learning in their schools may aid in creating an environment and culture where educators feel more able to use nature-based play and learning. Very few studies have investigated the enablers of nature-based play and learning; thus, the findings of this study provide important insight into potential methods for implementing nature-based play and learning into schools.

### 4.1. Strengths and Limitations

The strengths of this study include that most participants had more than 20 years’ experience and thus had rich and vast knowledge about education which they could draw upon. There was also equal representation of participants from rural and metropolitan schools, which allowed insight into the perspectives and experiences across economic and geographical contexts. However, despite these strengths, there were some limitations. The transferability of these findings may be limited as participants were self-selected from a group that expressed their interest in participating (through a previous survey). Thus, this research does not capture the perspectives and experiences of every principal and educator. Furthermore, only three participants represented disadvantaged schools. This limited representation of disadvantaged communities is typical of the literature, and more work is required to understand nature-based play and learning in disadvantaged communities. 

### 4.2. Implications for Future Research and Practice

It is recommended that mitigating barriers and promoting the enablers of nature-based play and learning becomes the focus of future research and practice with the aim of increasing uptake and practice to increase children’s exposure to the potential health and wellbeing benefits. In terms of practice, a lack of educator openness, knowledge and confidence can inhibit the use of nature-based play and learning. Educator openness, knowledge and confidence could be cultivated through training and a supportive environment will also play a role in cultivating this. Nature-based play and learning champions can play a role in creating this environment. Schools could focus on cultivating and nurturing champions within their community. Supportive leadership also plays an important role; schools can encourage leadership through training leadership staff in nature-based play and learning to enhance their understanding of the practice and its potential benefits for children. Promoting supportive leadership may also require significant cultural changes within the education system to change common perceptions around learning. These system-level cultural changes are also needed to help diminish the crowded curriculum and the perception that upper primary students should be learning exclusively in the classroom.

In terms of research, further research is needed to understand the practice of nature-based play and learning in disadvantaged communities. Future research is also required to investigate how the findings of this study can be applied to improve the implementation of nature-based play and learning. For example, research could investigate methods of effectively cultivating nature-based play and learning champions and promoting supportive leadership. Furthermore, the development of recommendations for implementing nature-based play and learning into primary schools may increase uptake and effective implementation. 

## 5. Conclusions

This study investigated educators’ perspectives and experiences of nature-based play and learning within the South Australian public primary school context. This research provides novel contributions to the evidence base in four key ways: (1) by investigating educators’ perspectives of nature-based play and learning in the Australian context; (2) by focusing on the perspectives of educators; (3) by investigating the enablers of nature-based play and learning; and (4) by doing so in the context of the COVID-19 pandemic. The findings highlight the positive perspectives of participants who described a variety of benefits and enablers to practice. However, participants also described practical barriers to practice. This study highlighted the growing interest in nature-based play and learning in South Australian primary schools. This suggests that now is the time to increase uptake by mitigating barriers and promoting the enablers of nature-based play and learning in order to provide children with more opportunities to experience the health and wellbeing benefits. It is recommended that this becomes the focus of future research and practice. 

## Figures and Tables

**Table 1:** The demographic characteristics of participants (n = 12) and their schools.

	n (%)
**Age group**	
18–24 years	0 (0)
25–34 years	1 (8)
35–44 years	3 (25)
45–54 years	5 (42)
55+ years	3 (25)
**Gender**	
Male	1 (8)
Female	10 (92)
**Role**	
Educator	8 (67)
Principal	2 (17)
Dual educator and principal	2 (17)
**Experience**	
0–5 years	1 (8)
6–10 years	1 (8)
11–15 years	1 (8)
16–20 years	2 (17)
20+ years	7 (58)
**Area**	
Rural	6 (50)
Metropolitan	6 (50)
**Index of Educational disadvantage**	
Unsure	0 (0)
1	1 (8)
2	1 (8)
3	1 (8)
4	2 (17)
5	2 (17)
6	4 (33)
7	1 (8)

## Data Availability

The data presented in this study are available within the article.

## Supplementary Materials

The following supporting information can be downloaded at: https://www.mdpi.com/article/10.3390/ijerph19063179/s1, Supplementary Materials S1: COREQ (COnsolidated criteria for REporting Qualitative research) Checklist; Supplementary Materials S2: Interview guide.
