# Supplementary material for: Primary School Educators’ Perspectives and Experiences of Nature-Based Play and Learning and Its Benefits, Barriers, and Enablers: A Qualitative Descriptive Study
Source: Int J Environ Res Public Health. 2022 Mar 8;19(6):3179. doi: 10.3390/ijerph19063179 (PMC8954476; doi:10.3390/ijerph19063179)
Supplement: Supplementary file 1 [file ijerph-19-03179-s001.zip › Supplementary Materials S2 Interview guide.pdf]

Interview guide

Overarching question: What are South Australian school staffs' perspectives and experiences of the benefits, barriers and enablers of nature-based play and learning?

**Topic**	**Prompts/ questions**
The practice of nature-based play and learning	How is nature-based play used at your school?<br>How is nature-based learning used at your school?<br>Who uses nature-based play and learning at your school?<br>What spaces are used for nature-based play and learning?<br>Are these spaces natural or purpose-built?
Benefits	Have you experienced any benefits of nature-based play and learning for your students/school/self? If so, what were they?
Barriers/ challenges	Have you experienced any consequences of using nature-based play and learning? If so, what were they?<br>Have you experienced any barriers to using nature-based play and learning? If so, what were they?<br>How have you overcome these barriers/ how could these barriers be overcome?
Enablers	What has enabled you to engage your students in nature-based play and learning?
Resources	Do you use the Nature Play SA Educators' Membership?<br>If so, is it useful? Why/ why not<br>Where do you get your inspiration and ideas for nature-based play and learning?<br>What would an ideal nature-based play and learning resource provide?
Recommendations	What is your advice for other schools who want to implement nature-based play and learning?
Wrap up	Is there anything else you would like to add?
